# The Composite of 3, 4-Dihydroxyl-Phenyl Lactic Acid and Notoginsenoside R1 Attenuates Myocardial Ischemia and Reperfusion Injury Through Regulating Mitochondrial Respiratory Chain

**DOI:** 10.3389/fphys.2021.538962

**Published:** 2021-07-12

**Authors:** Li Yan, Chun-Shui Pan, Yu-Ying Liu, Yuan-Chen Cui, Bai-He Hu, Xin Chang, Xiao-Hong Wei, Ping Huang, Jian Liu, Jing-Yu Fan, Quan Li, Kai Sun, Lu-Lu Yan, Ke He, Jing-Yan Han

**Affiliations:** ^1^Tasly Microcirculation Research Center, Peking University Health Science Center, Beijing, China; ^2^Department of Integration of Chinese and Western Medicine, School of Basic Medical Sciences, Peking University, Beijing, China; ^3^Key Laboratory of Stasis and Phlegm, State Administration of Traditional Chinese Medicine of the People’s Republic of China, Beijing, China; ^4^State Key Laboratory of Core Technology in Innovative Chinese Medicine, Tianjin, China; ^5^Beijing Microvascular Institute of Intergration of Chinese and Western Medicine, Beijing, China

**Keywords:** 3, 4-dihydroxyl-phenyl lactic acid, notoginsenoside R1, myocardium ischemia reperfusion injury, Complex I, Complex V

## Abstract

**Aim:**

3,4-Dihydroxyl-phenyl lactic acid (DLA) and notoginsenoside R1 (R1) are known to protect ischemia and reperfusion (I/R) injury by targeting Sirtuin1/NADH dehydrogenase (ubiquinone) 1 alpha subcomplex 10/the Mitochondrial Complex I (Sirt-1/NDUFA10/Complex I) and Rho-associated kinase/adenosine triphosphate (ROCK/ATP) ATP synthase δ subunit (ATP 5D), respectively. We hypothesized that a composite of the two may exhibit a more potent effect on I/R injury. The study was designed to test this hypothesis.

**Materials and Methods:**

Male Sprague–Dawley rats underwent left anterior descending artery occlusion and reperfusion, with or without DLA, R1, or a combination of 3,4-dihydroxyl-phenyl lactic acid and notoginsenoside R1 (DR) pretreatment. Heart function, myocardial morphology, myocardial infarct, myocardial blood flow (MBF), apoptosis, vascular diameter, and red blood cell (RBC) velocity in venules were evaluated. Myeloperoxidase (MPO), malondialdehyde (MDA), and 8-oxo-deoxyguanosine (8-OHdG) were assessed. The content of ATP, adenosine diphosphate (ADP), and adenosine monophosphate (AMP), the activity of mitochondrial respiratory chain Complex I and its subunit NDUFA10, the Mitochondrial Complex V (Complex V) and its subunit ATP 5D, Sirt-1, Ras homolog gene family, member A (RhoA), ROCK-1, and phosphorylated myosin light chain (P-MLC) were evaluated. R1 binding to Sirt-1 was determined by surface plasmon resonance.

**Results:**

DLA inhibited the expression of Sirt-1, the reduction in Complex I activity and its subunit NDUFA10 expression, the increase in MPO, MDA, and 8-OhdG, and apoptosis. R1 inhibited the increase in the expression of RhoA/ROCK-1/P-MLC, the reduction of Complex V activity and its subunit ATP 5D expression, alleviated F-actin, and myocardial fiber rupture. Both DLA and R1 reduced the myocardial infarction size, increased the velocities of RBC in venules, and improved MBF and heart function impaired by I/R. DR exhibited effects similar to what was exerted, respectively, by DLA and R1 in terms of respiratory chain complexes and related signaling and outcomes, and an even more potent effect on myocardial infarct size, RBC velocity, heart function, and MBF than DLA and R1 alone.

**Conclusion:**

A combination of 3,4-dihydroxyl-phenyl lactic acid and notoginsenoside R1 revealed a more potent effect on I/R injury via the additive effect of DLA and R1, which inhibited not only apoptosis caused by low expression of Sirt-1/NDUFA10/Complex I but also myocardial fiber fracture caused by RhoA/ROCK-1 activation and decreased expression of ATP/ATP 5D/Complex V.

## Introduction

Ischemic heart disease (IHD) accounts for the leading cause of death worldwide (Benjamin et al., 2018; Smit et al., 2019). Coronary artery bypass grafting and percutaneous coronary intervention have currently been applied for IHD. These strategies are able to restore myocardial perfusion, though, but frequently result in reperfusion injury (Yellon and Hausenloy, 2007), including reversible injury like myocardial stunning and arrhythmia, and irreversible injury like microvascular obstruction and death of cardiomyocytes (Hausenloy and Yellon, 2013). Up to now, no effective management is available for protecting against myocardial reperfusion injury.

Ischemia and reperfusion (I/R) injury is a complex process with the mitochondrial respiratory chain as a central player. I/R injury initiates with ischemia, during which the oxygen and nutrient supply are deprived, leading to the Mitochondrial Complex V (Complex V) [adenosine triphosphate (ATP) synthase] dysregulation resulting in a decline of ATP production (Rouslin, 1983) and diversity of insults, such as actin cytoskeleton depolymerization (Shi et al., 2009) and disruption of intercellular junctions (Abdullahi et al., 2018). Thus, substances that act at Complex V have been proposed as an option to deal with ischemia reperfusion (I/R) injury (He et al., 2014). Subsequent reperfusion imposes an oxygen surge on the Mitochondrial Complex I (Complex I), which triggers the generation of reactive oxygen species (ROS) (Balaban et al., 2005), provoking myocardial injury and cardiomyocyte death through a number of mechanisms, including the opening of mitochondrial permeability transition pore (mPTP), Ca^2+^ overload, neutrophil infiltration, etc. (Kalogeris et al., 2016). Hence, interventions targeting Complex I and its subunits such as NADH dehydrogenase (ubiquinone) 1 alpha subcomplex 10 (NDUFA10) or its upstream regulator Sirtuin1 (Sirt-1) are a promising strategy for protecting against oxidative stress after I/R (Yang et al., 2015). In addition, increasing evidence shows that Ras homolog gene family, member A (RhoA) and its main effector Rho-associated kinase (ROCK) are implicated in extensive process in cardiac muscle cells (Cai et al., 2016), including the organization of the actin cytoskeleton, cell contraction, adhesion, apoptosis, and the like (Takai et al., 1995), the manifestations that may be impaired by I/R injury, suggesting the essential role of RhoA/ROCK in the pathogenesis of I/R injury. Taken together, the available evidence demonstrated the implication of both Sirt-1/NDUFA10/Complex I and RhoA/ROCK-1/Complex V in I/R injury. Therefore, to cope with cardiac I/R injury, an option with the potential to regulate Sirt-1/NDUFA10/Complex I and RhoA/ROCK-1/Complex V simultaneously is urgently needed.

The compound Chinese medicines Cardiotonic Pills^®^ and QiShenYiQi Pills^®^ both contain *Salvia miltiorrhiza* and notoginseng as ingredients and are widely used in China for the prevention and management of coronary heart disease, angina, and myocardial I/R injury (Zhao et al., 2010; Han et al., 2019). Cardiotonic Pills^®^ has already passed the FDA phase II clinical trials for treatment of coronary heart disease and angina pectoris and is currently undergoing phase III clinical trials. Our study confirmed that both compounds improved myocardial I/R injury (Zhao et al., 2010, Lin et al., 2013). 3,4-Dihydroxyl-phenyl lactic acid (DLA) is a water-soluble compound derived from *Salvia miltiorrhiza*. A range of cardiovascular protective effects has been reported for DLA, including antioxidation, anti-apoptosis, vasodilation, inflammation regulation, lipidemia control, etc., which are known to implicate multiple signalings such as PI3K/Akt-ERK1/2/Nrf2/HO-1, Bcl-2/Bax, and eNOS (Zhang et al., 2019). Our lab demonstrated that DLA could bind to and activate Sirt-1, protect I/R-induced decrease in NDUFA10 expression, ameliorate Complex I activity and mitochondrial function, and diminish ROS generation and myocardial I/R injury in rats (Yang et al., 2015). However, it is unclear as yet whether or not DLA can regulate the RhoA/ROCK pathway to improve ATP synthase δ subunit (ATP 5D) expression and ATP content. Notoginsenoside R1 (R1) is the main active component isolated from *Panax notoginseng*. Studies demonstrated that R1 mitigated the infarct size and elevated the cardiomyocyte viability to restrain myocardial I/R injury by a variety of mechanisms, including anti-inflammatory (Xia et al., 2015), antioxidation (Yu et al., 2016), anti-apoptosis (Yu et al., 2016), and improvement of energy metabolism (He et al., 2014). A previous study in our lab confirmed that R1 could inhibit ROCK and enhance mitochondrial ATP 5D expression contributing to its protective effect on cardiac I/R injury (He et al., 2014). However, it is so far unknown whether or not R1 is able to protect Sirt-1/NDUFA10/Complex I from downregulation after I/R and resultant oxidative stress and apoptosis.

In view of the complexity of the pathogenesis of I/R injury and the diverse targets the DLA and R1 act at, we speculated that a combination of DLA and R1 might be more efficient than DLA and R1 either alone in attenuating myocardial I/R injury. The current work was to test this hypothesis and the rationale behind it.

## Materials and Methods

### Animals

Male Sprague–Dawley (SD) rats (weight: 230–270 g) were purchased from the Animal Center of Peking University [Certificate No. SCXK (Jing) 2006–0008]. The animals were fed with a standard laboratory diet and water and were subjected to fasting for 12 h prior to the experiment. The experimental protocols were ratified by Peking University Biomedical Ethics Committee Experimental Animal Ethics Branch (LA2010-001), complying with the Guide of Peking University Animal Research Committee.

### Drugs

3,4-dihydroxyl-phenyl lactic acid and R1 (purity ≥ 99.9%) were provided by Fengshanjian Medicine Research Co. Ltd. (Kun Ming Feng-Shan-Jian Medical Company, Yunnan, China) and were dissolved in normal saline at a concentration of 1.25 mg/ml as stock solutions.

### Rat Cardiac I/R Modeling and Treatment

Animals were anesthetized using 2% phenobarbital sodium (60 mg/kg) by peritoneal injection and restrained in a dorsal position and, at the same time, inserted with an animal breathing apparatus through the mouth. After thoracotomy, the left anterior descending coronary artery of the heart was ligated by a 5-0 suture silk for 30 min for induction of ischemia and then unlashed for reperfusion. The animals in the NS + Sham group underwent the same procedure but without the ligation of suture silk. Thirty minutes before ischemia, the animals in DLA, R1, and DR pretreatment groups received DLA (4 mg/kg/h), R1 (1 mg/kg/h), and DR (4 mg DLA + 1 mg R1/kg/h), respectively, by continuous infusion through the femoral vein. The proportion of DLA and R1 in the DR group was determined based on the formula of Cardiotonic Pills^®^ and a pilot experiment. The rats in the NS + Sham group and the NS + I/R group were given normal saline the same way.

### Assessment of Infarct Size

Rat hearts were isolated 90 min after reperfusion and cut transversely into five slices (1 mm thick) in between the apex cordis and the ligation site. Slices were stained by a 0.375% solution of 2,3,5-triphenyltetrazolium chloride (TTC) (Sigma, St. Louis, MO, United States) at 37°C for 15 min and pictured by Digital sight (DS-5M-U; Nikon, Nanjing, China), with the infarction zone displaying as white, the area at risk (AAR) as pink, and the non-infarction zone as blue. Image-Pro Plus 6.0 (Media Cybernetic, Bethesda, MD, United States) was applied to determine the infarct size, the AAR, and the left ventricular (LV) size in each slice. The averages of AAR/LV and infarct area/AAR from five slices were used to denote the extent of myocardial infarction.

### Determination of Myocardial Blood Flow

Myocardial blood flow (MBF) was detected following thoracotomy using a Laser-Doppler Perfusion Imager (PeriScan PIM3 System; PERIMED, Stockholm, Sweden) at baseline, 30 min post-ischemia, and 30 and 60 min postreperfusion, respectively. A computer-controlled optical scanner head was positioned 18 cm from the exposed heart, with the beam irradiating the tissue 0.5 mm deep. The resulting color-coded images were assessed using the software LDPIwin 3.1 (PeriScan PIM3 System; PERIMED, Stockholm, Sweden), with blue to red indicating a low to high value of MBF. The ratio of the value of MBF at a time point to baseline was presented as a score of MBF at that time point.

### Determination of Red Blood Cell Velocity and Diameter of Microvessels

The heart continuously underwent surface perfusion with a saline solution of 37°C. Coronary microvessels with a diameter of 25–40 μm were selected and examined under an upright microscope (BX51WI, Olympus, Olympus, Tokyo, Japan) equipped with a high-speed video camera (APX, Photon Fastcam-ultimate, Tokyo, Japan). Images were acquired and displayed on a monitor (20PF5120, Philips, Amsterdam, Netherlands) and recorded by a DVD videocassette recorder (DVR-560H, Philips, Tokyo, Japan). The assessment of RBC velocity in venule was accomplished by recording for 4 s at 500 frames/s, replayed at 25 frames/s and measured using the Image-Pro Plus 5.0 software (Media Cybernetic, Bethesda, MD, United States). The ratio of the value at a time point to baseline was calculated as the score for that time point. The diameter of the vessels was detected by Image-Pro Plus 6.0 (Media Cybernetic, Bethesda, MD, United States).

### Heart Function Test

Heart function was tested by a bio-function experiment system BL-420F (Chengdu Taimen Technology Ltd., Chengdu, Sichuan, China), which was connected to cannulation inserted into the LV through the right carotid artery. Heart rate (HR), left ventricular systolic pressure (LVSP), left ventricular developed pressure (LVDP), left ventricular maximum upstroke velocity (+dp/dtmax), and left ventricular maximum descent velocity (–dp/dtmax) were evaluated at the time points indicated.

### Myocardial Histological Analysis and Immunohistochemistry

At 90 min after reperfusion, the heart tissue was removed from the middle one-third between the apex and the ligation point, fixed in 4% paraformaldehyde (PFA) solution for 48 h, and prepared for paraffin sections. Paraffin sections (5 μm) for hematoxylin and eosin (HE) staining were processed using standard approaches and observed by a light microscope. A pathologist unaware of the experiments evaluated I/R injures including myocardial fiber rupture, myocardial interstice edema, and leukocyte infiltration based on the criteria of five grades: (0) none; (1) weak; (2) moderate; (3) strong; and (4) very strong (Du et al., 2020). The samples for immunohistochemistry were dewaxed, rehydrated, and incubated with primary antibody against myeloperoxidase (MPO, 1:200, Thermo Scientific, CA, United States) overnight at 4°C after being blocked with bovine serum albumin, and then revealed by HRP-conjugated secondary antibody and a DAB Substrate Kit. Photomicroscopy was then performed in penumbra at ×200 magnification using a microscope (BX512DP70, Olympus, Tokyo, Japan).

### Filamentous Actin and TUNEL Staining

Some paraffin sections underwent filamentous actin (F-actin) and terminal deoxynucleotidyl transferase-mediated dUTP-biotin nick end-labeling (TUNEL) double staining, wherein F-actin was stained by rhodamine phalloidine (R415; Invitrogen, Carlsbad, CA, United States), and TUNEL staining was conducted by a cell death detection kit (Roche, Basel, Switzerland) following the instruction of the manufacturer. The nuclei were labeled with Hoechest33342. Five fields were randomly selected in penumbra in each section, examined with a Laser Scanning Confocal Microscope (TCS SP5; Leica, Mannheim, Germany), and the TUNEL positive cells were counted, the average of which was expressed as the cell number per field.

### Electron Microscopy

At 90 min after reperfusion, the hearts were perfused by 3% glutaraldehyde (Ted Pella, Redding, CA, United States) for 40 min. Cardiac tissue was excised from the penumbra of the left ventricle as described previously (Zhao et al., 2010), trimmed into blocks <2 mm^3^, and fixed in 3% glutaraldehyde at 4°C overnight. Following washing three times with 0.1 mol/L phosphate-buffered solution, the samples were post-fixed with 1% osmium tetraoxide for 2 h and processed for thin sectioning. The thin sections were examined with an electron microscope (JEM 1400 plus, JEOL, Tokyo, Japan).

### Assessment of Energy Metabolism

The hearts were isolated after 90-min reperfusion. The tissues of the penumbra areas were dissected and stored at –80°C before use. The protein extraction was carried out using a protein extraction kit (Applygen Technologies, Beijing, China), as per the instruction of the manufacturer. Briefly, the tissue was subjected to homogenization, incubation on ice for 30 min, and centrifugation at 20,000*g*, 4°C, for 30 min, taking the supernatant as the whole protein. ELISA was applied to assess the content of ATP, adenosine diphosphate (ADP), and adenosine monophosphate (AMP) of the myocardium, and the result was read by a microplate reader (Multiskan MK3, Thermo, San Jose, CA, United States) as per the instruction of the manufacturer.

### Assessment of MPO, MDA, and 8-OHdG Level in Myocardial Tissue

The level of myeloperoxidase (MPO), malondialdehyde (MDA), and 8-oxo-deoxyguanosine (8-OHdG) in the penumbra of the left ventricle was determined as indicators of tissue peroxidation by using a respective ELISA kit (GBD Ltd., CA, United States) as per the instruction of the manufacturer.

### Detection of Complex I and Complex V Activity

The heart sample from the penumbra was incubated with detergent on ice for 30 min. Following lysis by RIPA lysate and centrifugation at 20,000*g* for 20 min, the supernatant was harvested and subjected to protein concentration determination with a BCA protein assay kit (Applygen Technologies, Beijing, China) in the light of the instruction of the manufacturer. Complex I activity was determined by a Complex I Enzyme Activity Microplate Assay Kit (Abcam, Cambridge, United Kingdom). The absorbance was assessed at 450 nm for 30 min at room temperature using a kinetic program. Complex V activity was measured using an ATP Synthase Enzyme Activity Microplate Assay Kit (Abcam, Cambridge, United Kingdom), with the plate set in the microplate reader (Multiskan MK3, Thermo, San Jose, CA, United States). The absorbance was assessed at 340 nm, 30°C for 60 min using a kinetic program. The absorbance change (in optical density units/min) was used to express the activity.

### Western Blot

The mixture of whole protein and 5× electrophoresis sample buffer was subjected to electrophoresis on 10 or 12% SDS-PAGE. The separated proteins were transferred to a polyvinylidene difluoride membrane. Following blocking with 5% defatted milk powder, rinsing with TBS-Tween, the membrane was cut and incubated overnight at 4°C with antibodies against GAPDH (1:4,000, CST, VT, United States), Caspase 3 (1:1,000, CST, VT, United States), cleaved Caspase 3 (1:1,000, CST, VT, United States), Caspase 9 (1:1,000, CST, VT, United States), cleaved Caspase 9 (1:1,000, CST, VT, United States), Bcl2 (1:1,000, CST, VT, United States), Bax (1:2,000, CST, VT, United States), ATP 5D (1:1,000, Abcam, Cambridge, United Kingdom), RhoA (1:1,000, Abcam, Cambridge, United Kingdom), ROCK-1 (1:1,000, Abcam, Cambridge, United Kingdom), phosphorylated myosin light chain (P-MLC) (1:1,000, CST, VT, United States), Sirt-1 (1:800, CST, VT, United States), and NDUFA10 (1:2,000, Santa Cruz, CA, United States). After rinsing, the membrane was incubated with HRP-conjugated secondary antibody (1:5,000, Cell Signaling Technology, VT, United States) for 1 h at room temperature. The target protein was quantified by scanning densitometry with a bio-image analysis system (Image-Pro plus 6.0; Media Cybernetic, Bethesda, MD, United States).

### Surface Plasmon Resonance

Biacore T200 (Biacore, GE Healthcare, Uppsala, Sweden) was used for docking a carboxymethylated 5 (CM5) sensor chip (GE Healthcare Life Sciences, London, United Kingdom) on which 40 μl of human Sirt-1 full-length protein (Abcam, Cambridge, MA, United States) (1 μg/μl in 10 mM sodium acetate, pH 4.5) was immobilized by injection at a rate of 5 μl/min. R1 (2,000 μM in a running buffer) was diluted into different concentrations and injected at 30 μl/min over the proteins and a control sensor chip from low to high concentration to avoid artifacts. The equilibrium dissociation constant (KD) was determined by fitting a 1:1 Langmuir model using the Biacore T200 evaluation software v2.0 (Biacore, GE Healthcare, Uppsala, Sweden).

### Statistical Analysis

Data were presented as mean ± SEM. Statistical test was conducted by one-way ANOVA followed by the Newman–Keuls test or using two-way ANOVA followed by Bonferroni for multiple comparisons [myocardial blood flow (MBF), venules diameter, and red blood cell velocity (RBC) velocity]. Data were analyzed by virtue of the GraphPad Prism 5 software. A *p* value < 0.05 was regarded as statistically significant.

## Results

### DLA, R1, or DR Diminishes Infarct Size in Rat Heart After I/R

**FIGURE 1:**
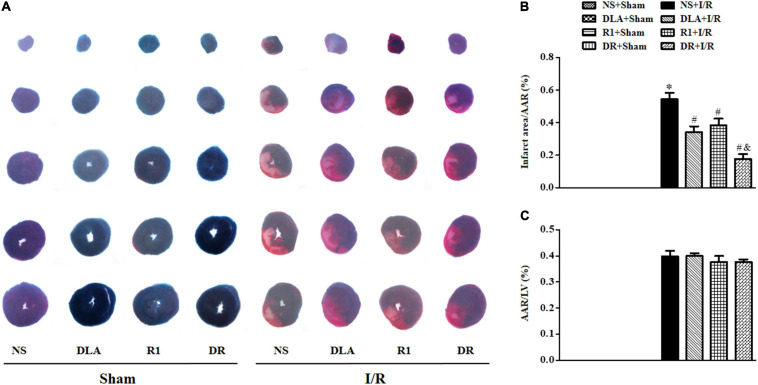
The effect of 3,4-dihydroxyl-phenyl lactic acid (DLA), notoginsenoside R1 (R1), and a combination of 3,4-dihydroxyl-phenyl lactic acid and notoginsenoside R1 (DR) pretreatment on the myocardial infarct of rats subjected to ischemia reperfusion (I/R) for 90 min. **(A)** Example slice of 2,3,5-triphenyltetrazolium chloride (TTC)-stained left ventricle in the NS + Sham, DLA + Sham, R1 + Sham, DR + Sham, NS + I/R, DLA + I/R, R1 + I/R, and DR + I/R groups. The myocardium infarct is revealed as pale white. **(B,C)** The quantitative analysis of area at risk/left ventricular (AAR/LV) and infarct area/AAR 90 min after I/R in the different groups. Data are mean ± SEM (n = 6). *p < 0.05 vs. NS + Sham group; #*p* < 0.05 vs. NS + I/R group; &*p* < 0.05 vs. DLA + I/R and R1 + I/R group.

Myocardial infarct was detected by TTC staining at 90 min after reperfusion to evaluate the efficiency of different options. Displayed in Figure 1A are the representative pictures of heart slices in different groups, wherein the pink area indicates ischemic tissue, while the white indicates the infarction region. As noticed, no infarct was present in the slices in the NS + Sham, DLA + Sham, R1 + Sham, and DR + Sham groups. On the contrary, prominent infarct areas were observed in slices of the NS + I/R group. As compared to the NS+I/R group, slices from the DLA, R1, and DR pretreatment groups revealed less infarction while similar ischemic regions. A quantification of AAR/LV and infarct area/AAR further confirms the result (Figure 1B,C), suggesting that DLA, R1, and DR exert protective effects on I/R-induced myocardium infarct. Intriguingly, DR showed more effectiveness than R1 and DLA in reducing the area of infarction.

### DR Prevents the Reduction of MBF in Rat Hearts Following I/R

**FIGURE 2:**
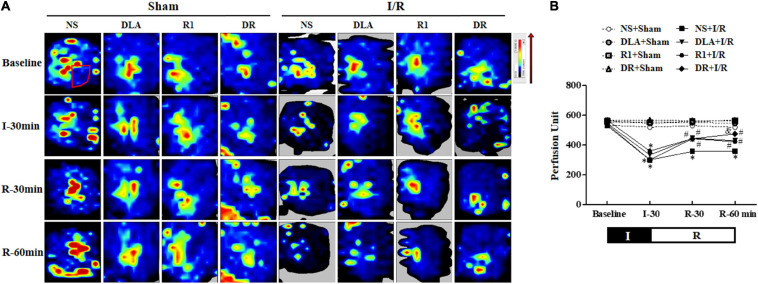
The effect of DLA, R1, and DR pretreatment on myocardial blood flow (MBF) of rats after I/R. **(A)** The MBF images acquired by a Laser-Doppler Perfusion Imager in the NS + Sham, DLA + Sham, R1 + Sham, DR + Sham, NS + I/R, DLA + I/R, R1 + I/R, and DR + I/R groups at baseline and 0, 30, and 60 min after reperfusion. Flux is represented by a color scale (from dark blue to red denoting low to high flux). The area within the box showed the location of the left ventricle where the blood flow was detected and quantified. **(B)** Time course of changes in MBF in different conditions. Data are mean ± SEM (n = 6). I, ischemia phase; R, reperfusion phase. *p < 0.05 vs. NS + Sham group; #*p* < 0.05 vs. NS + I/R group; &*p* < 0.05 vs. DLA + I/R group and R1 + I/R group.

Myocardial blood flow was determined by the Laser Scanning Doppler at a different time point with the representative images of each group displayed in Figure 2A. The MBF of the eight groups at baseline showed no apparent difference. A noticeable decline in MBF was observed in the NS + I/R group at 30 min post-ischemia, which sustained over the observation. DLA, R1, and DR pretreatment prevented the decrease in MBF after I/R at 30- and 60-min reperfusion.

Figure 2B shows how MBF changed with time in the eight groups, which verified the survey of Figure 2A. Specifically, the MBF in the NS + I/R group was down to about 50% of baseline after ischemia, which did not recover until the 60-min reperfusion. MBFs in DLA, R1, and DR pretreatment groups changed with time in a way similar to the NS + I/R group, with no difference over the period of ischemia, while with apparent recovery after reperfusion. The result indicates that DR pretreatment was more effective than R1 or DLA alone.

### DLA or DR Attenuates the Decrease in RBC Velocity by I/R

**FIGURE 3:**
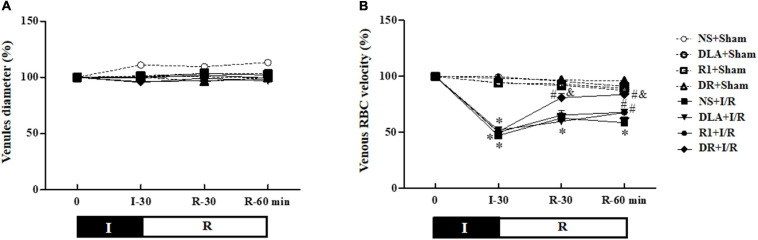
The effect of DLA, R1, and DR pretreatment on the diameter of vessels and red blood cell velocity’s (RBC) velocity in coronary venules in rats subjected to I/R. **(A)** The time course of change in the diameter of vessels. **(B)** The time course of change in RBC’s velocity in coronary venules. Data are mean ± SEM (n = 6). I, ischemia phase; R, reperfusion phase. *p < 0.05 vs. NS + Sham group; #*p* < 0.05 vs. NS + I/R group; &*p* < 0.05 vs. DLA + I/R group and R1 + I/R group.

Figure 3A shows the time course of the change in diameters of the coronary microvessels in the eight groups, which revealed no significant difference among the groups over the period of examination. This result indicated that the vasodilation performance of coronary microvessels was affected neither by the experimental protocol nor by the medicine treatment.

In the beating heart, RBCs moving inside the coronary microvessels were watched with a microscope equipped with a high-speed video camera, and the time course of the RBC velocity change in coronary venules is depicted in Figure 3B. In the NS + Sham, DLA + Sham, R1 + Sham, and DR + Sham groups, the RBC velocity in venules remained nearly constant over the observation period. In the NS + I/R group, the RBC velocity significantly slowed after ischemia, which was kept at a relatively low level by 60 min of reperfusion. DLA and R1 elevated the RBC velocity at 60 min after reperfusion, while pretreatment with DR significantly recovered the RBC velocity starting from 30 min after reperfusion. DR pretreatment again showed better effectiveness compared to DLA or R1 alone.

### DR Ameliorates the Impairment of Heart Function Induced by I/R

**FIGURE 4:**
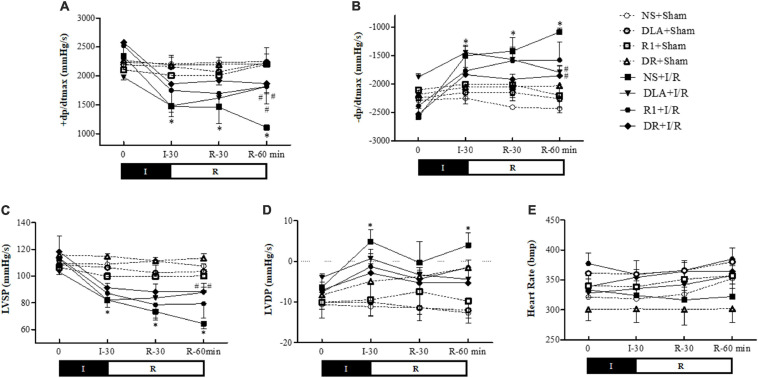
The effect of DR pretreatment on the rat heart function. Presented are the time courses of +dP/dtmax **(A)**, –dP/dtmax **(B)**, left ventricular systolic pressure (LVSP) **(C)**, left ventricular diastolic pressure (LVDP) **(D)**, and heart rate (HR) **(E)**, in different groups. The linear mixed-effect models were analyzed for repeated measurement data, and least-squares means were calculated between the groups at different time points. Values are means ± SEM (*n* = 6). I, ischemia phase; R, reperfusion phase. *p < 0.05 vs. NS + Sham group; #*p* < 0.05 vs. NS + I/R group.

The heart function of rats in different groups was determined. The results (Figure 4) showed that in comparison with the NS + Sham group, ischemia for 30 min caused a significant decline in LVSP and +dP/dtmax, and elevation in LVDP and –dP/dtmax, indicating an impairment of the heart function. Reperfusion led to a further decline in +dP/dtmax as well as a significant decrease in LVSP and a sustained increase in –dP/dtmax and LVDP. Evidently, the changes in +dP/dtmax, –dP/dtmax, and LVSP by I/R were ameliorated by pretreatment with DLA and DR at the end of reperfusion, while R1 at the present dose only improved the cardiac function +dP/dtmax. DLA, R1, and DR had no effect on LVDP. In addition, no significant difference was observed among the group over the observation.

### DLA, R1, or DR Diminishes Myocardial Injury by I/R

**FIGURE 5:**
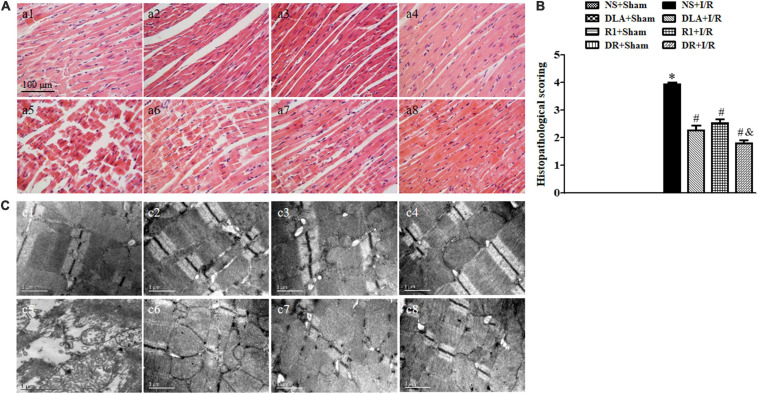
The effect of DLA, R1, and DR pretreatment on myocardial histology and ultrastructure in rats exposed to I/R for 90 min. **(A)** Examples of HE-stained histological photographs of myocardium in the NS + Sham group **(a1)**, the DLA + Sham group **(a2)**, the R1 + Sham group **(a3)**, the DR + Sham group **(a4)**, the NS + I/R group **(a5)**, the DLA + I/R group **(a6)**, the R1 + I/R group **(a7),** and the DR + I/R group **(a8)**. Bar = 100 μm. **(B)** Histological analysis of hematoxylin-eosin (HE) staining. Data are mean ± SEM (n = 3). *p < 0.05 vs. NS + Sham group; #*p* < 0.05 vs. NS + I/R group; &*p* < 0.05 vs. DLA + I/R group and R1 + I/R group. **(C)** Examples of electron micrographs from the NS + Sham group **(c1)**, the DLA + Sham group **(c2)**, the R1 + Sham group **(c3)**, the DR + Sham group **(c4)**, the NS + I/R group **(c5)**, the DLA + I/R group **(c6)**, the R1 + I/R group **(c7),** and the DR + I/R group **(c8)**.

**FIGURE 6:**
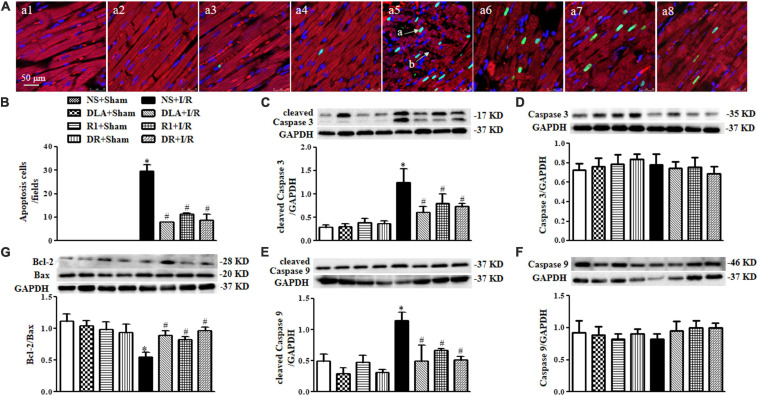
The effect of DLA, R1, and DR pretreatment on myocardial apoptosis and filamentous actin (F-actin) structure in rats after I/R for 90 min. **(A)** Examples of myocardium photographs showing F-actin and TUNEL-positive apoptotic cells in the NS + Sham group **(a1)**, the DLA + Sham group **(a2)**, the R1 + Sham group **(a3)**, the DR + Sham group **(a4)**, the NS + I/R group **(a5)**, the DLA + I/R group **(a6)**, the R1 + I/R group **(a7),** and the DR + I/R group **(a8)**. Nuclei are stained as blue, F-actin red, and terminal deoxynucleotidyl transferase-mediated dUTP-biotin nick end-labeling (TUNEL)-positive cells green (*arrow a*: TUNEL-positive cell; *arrow b*: disrupture of F-actin). Bar = 50 μm. **(B)** Quantitative assessment of apoptotic cardiomyocytes in various conditions. **(C)** Examples of Western blot bands and densitometric values of cleaved Caspase 3 corrected for GAPDH. **(D)** Examples of Western blot bands and densitometric values of Caspase 3 corrected for GAPDH. **(E)** Examples of Western blot bands and densitometric values of cleaved Caspase 9 corrected for GAPDH. **(F)** Examples of Western blot bands and densitometric values of Caspase 9 corrected for GAPDH. **(G)** Western blot result of Bcl-2/Bax in the myocardium (*n* = 6). Data are expressed as means ± SEM (n = 6). *p < 0.05 vs. NS + Sham group; #*p* < 0.05 vs. NS + I/R group.

To assess the effect of DR pretreatment on the myocardium structure after I/R, the histological scoring of the myocardium in different groups was first examined (Figure 5A,B). In the NS + I/R group, apparent alterations arose in the penumbra around the infarcted zone of myocardial tissues as compared with the Sham groups, including myocardial fibers rupture, edema of myocardial interstice, and leukocyte infiltration. I/R challenge significantly injured the myocardial tissue with a histopathological score of around 4. Noticeably, pretreatment with DLA, R1, and DR significantly reduced histopathological scores, with DR being more effective. The results of F-actin labeled by rhodamine phalloidin in Figure 6A further validated the above results. Obviously, the I/R-evoked F-actin decline and rupture were prevented by pretreatment with DLA, R1, or DR. The representative ultrastructural pictures of myocardium in the eight groups are displayed in Figure 5C. In the NS + Sham, DLA + Sham, R1 + Sham, and DR + Sham groups, the myocardium presented regularly arranged myofibrils and sarcomeres with well-preserved mitochondria. The I/R brought about a considerable injury in the myocardium ultrastructure, exhibiting disrupted myofibrils and swelling mitochondria. This injury was alleviated by pretreatment with DLA, R1, or DR. Taken together, pretreatment with DLA, R1, and DR diminished the myocardium injury induced by I/R.

### DLA, R1, or DR Attenuates Myocardial Cell Apoptosis Following I/R

To address the role of DLA, R1, and DR in myocardial cell apoptosis, double staining of F-actin and TUNEL was undertaken for the penumbra around infarction areas from eight groups. The representative images are displayed in Figure 6A, with nuclei staining blue, F-actin red, and TUNEL positive cells’ nuclei green. Compared with the Sham groups, the myocardium in the I/R group displayed ruptured myocardial fibers and F-actin and a large number of TUNEL positive cells. These alterations were relieved by pretreatment with DLA, R1, or DR. The statistical result of the percent of TUNEL positive cardiomyocytes in penumbra around the infarction zone further confirmed the above results (Figure 6B). Apoptosis is regulated by apoptotic proteins; hence, we detected the level of cleaved Caspase 3, cleaved Caspase 9, Caspase 3, Caspase 9, Bcl-2, and Bax in whole-heart lysates from various groups using Western blot (Figure 6C–G). The results demonstrated that DLA, R1, or DR prevented the increase in cleaved Caspase 3 and cleaved Caspase 9 after I/R (Figure 6C,E). Furthermore, the ratio of Bcl-2/Bax was noticeably decreased by I/R injury, which was protected by pretreatment with DLA, R1, and DR (Figure 6G). These results demonstrated the antiapoptotic activity of DLA, R1, and DR.

### DLA, R1, or DR Reduces Oxidative Stress Following I/R

**FIGURE 7:**
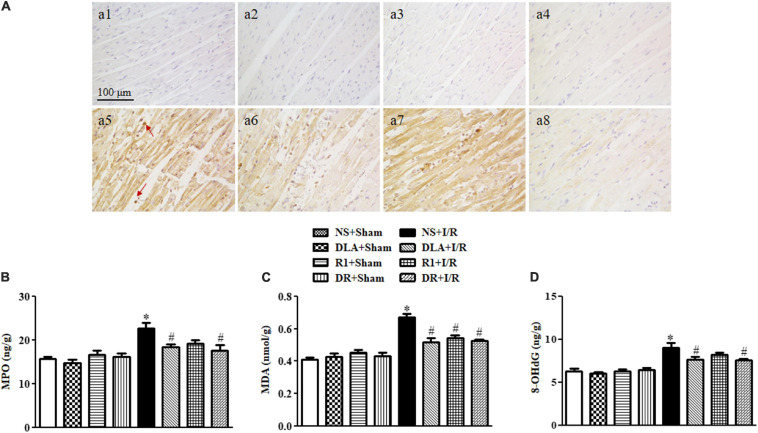
The effect of DLA, R1, and DR pretreatment on myeloperoxidase (MPO), malondialdehyde (MDA), and 8-oxo-deoxyguanosine (8-OHdG) level in the myocardial tissue of rats after I/R for 90 min. **(A)** Representative photographs of immunohistochemistry staining for MPO of myocardium in the NS + Sham group **(a1)**, the DLA + Sham group **(a2)**, the R1 + Sham group **(a3)**, the DR + Sham group **(a4)**, the NS + I/R group **(a5)**, the DLA + I/R group **(a6)**, the R1 + I/R group **(a7),** and the DR + I/R group **(a8)**. Bar = 100 μm. Arrows indicate MPO-positive cells. **(B)** Quantitative analysis of the MPO level by ELISA in rat myocardium in different groups (*n* = 6). **(C)** Quantitative analysis of the MDA level by ELISA in rat myocardium in different groups (*n* = 6). **(D)** Quantitative analysis of the 8-OHdG level by ELISA in rat myocardium in different groups (*n* = 6). Data are expressed as means ± SEM (n = 6). *p < 0.05 vs. NS + Sham group; #*p* < 0.05 vs. NS + I/R group.

In view of the close association between apoptosis and oxidative stress, we next detected the level of MPO, MDA, and 8-OHdG, the molecules relevant to oxidative stress, in penumbra around infarction areas at 90 min reperfusion. As shown in Figure 7A, many MPO positive neutrophils occurred in the myocardium in the I/R group, which was decreased significantly by pretreatment with DLA and DR. The ELISA assessment of MPO (Figure 7B) verified the result above. Akin to MPO, MDA production was enhanced by I/R, which was protected by DLA, R1, or DR pretreatment (Figure 7C). Likewise, I/R evoked an increase in the 8-OHdG level, which was relieved by DLA and DR pretreatment with significance (Figure 7D).

### DLA or DR Alleviates the Downregulated Expression of Sirt-1 and NDUFA10 and Reduced Complex I Enzyme Activity After I/R

**FIGURE 8:**
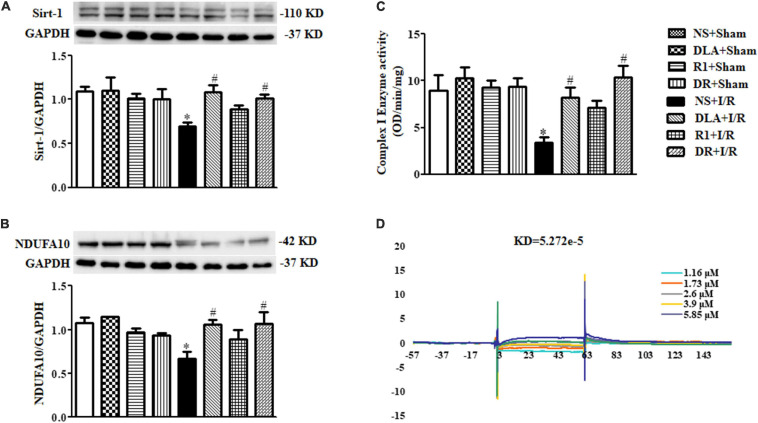
3,4-dihydroxyl-phenyl lactic acid and DR, but not R1, bind to and activate Sirtuin1 (Sirt-1). **(A)** Displayed are examples of Western blot bands of Sirt-1 in various groups, with quantitative results shown below. Quantitative results for Sirt-1 band intensities were normalized to GAPDH (n = 6). **(B)** Representative Western blot bands of NADH dehydrogenase (ubiquinone) 1 alpha subcomplex 10 (NDUFA10) in various groups with quantitative results shown below. Quantitative results for NDUFA10 band intensities were normalized to GAPDH (n = 6). **(C)** Complex I activity determined based on the slope of increase in OD at 450 nm in all groups (n = 6). Data are expressed as means ± SEM (n = 6). *p < 0.05 vs. NS + Sham group; #*p* < 0.05 vs. NS + I/R group. **(D)** Examples of sensorgrams obtained for R1 at 1.16, 1.73, 2.6, 3.9, and 5.85 μM (curves from bottom to top) using surface plasmon resonance (SPR).

Mitochondria are known to contribute to both oxidative damage and apoptosis in I/R injury. Our lab has reported that DLA can bind to Sirt-1 to preserve the expression of NDUFA10 and Complex I activity (Yang et al., 2015). We thus speculated that DR, a mixture of DLA and R1, may exhibit the same potential in the present case. The results in Figure 8A–C verified this hypothesis, showing that DLA and DR both ameliorated the decrease in Sirt-1 and NDUFA10 expression and enhanced Complex I enzyme activity after I/R, while R1 had no effect. The failure of R1 to attenuate the downregulated Sirt-1 and NDUFA10 expression and Complex I activity after I/R is likely due to its inability to bind to Sirt-1 (Figure 8D).

### DLA, R1, and DR Regulate Energy Metabolism Via Different Pathways

**FIGURE 9:**
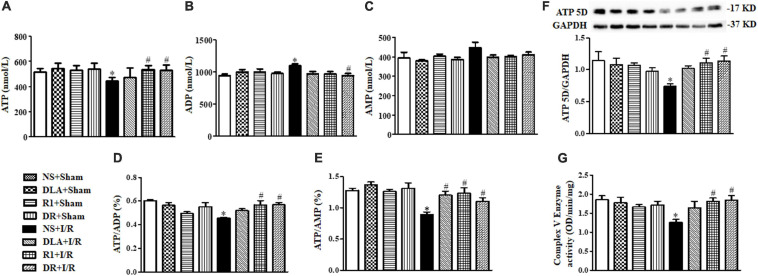
The effect of DLA, R1, and DR pretreatment on myocardial energy metabolism, the Mitochondrial Complex V (Complex V) enzyme activity, and ATP 5D expression in rats after I/R for 90 min. **(A–C)** Quantitative evaluation of adenosine triphosphate (ATP), adenosine diphosphate (ADP), and adenosine monophosphate (AMP) by ELISA in myocardial tissue. **(D and E)** Quantitative evaluation of ATP/ADP and ATP/AMP in myocardial tissue. Data are expressed as means ± SEM (n = 6). *p < 0.05 vs. sham group, #*p* < 0.05 vs. I/R group. **(F)** Representative Western blot bands of ATP 5D in various groups with quantitative results shown below. Quantitative results for ATP 5D band intensities were normalized to GAPDH (n = 6). **(G)** Complex V activity determined according to the slope of increase in OD at 450 nm in all groups (n = 6). Data are expressed as means ± SEM (n = 6). *p < 0.05 vs. NS + Sham group; #*p* < 0.05 vs. NS + I/R group.

Ruptured F-actin observed after the I/R challenge suggested an impaired energy metabolism since F-actin polymerization depends on ATP supply. We thus detected the content of ATP, ADP, and AMP in various groups by ELISA. As noticed in Figure 9, the I/R group revealed a significant decrease in ATP **(A)**, ATP/ADP **(D)**, and ATP/AMP **(E)**, and an increase in ADP **(B)**, as compared with the sham groups, while there was no obvious change in AMP **(C)**, hinting at the dysregulation of ATP metabolism after I/R. The decrease in ATP was restored by DR as well as by R1 or DLA alone. Pretreatment with R1 and DR significantly restrained both ATP/ADP and ATP/AMP from the decrease by I/R, while DLA only restored ATP/AMP but not ATP/ADP, suggesting that R1 and DR are more efficient in regulating ATP metabolism than DLA alone. To gain insight into the rationale behind this phenomenon, we detected Complex V activity and the level of ATP 5D, a subunit of Complex V, in different conditions. The activity of Complex V was reduced significantly by I/R, as compared to that of the sham groups, which was significantly prevented by pretreatment with R1 and DR, but not DLA (Figure 9G). Likewise, the level of ATP 5D was decreased by I/R, which was restored by pretreatment with R1 and DR, but not DLA (Figure 9F). This result implies that DLA regulated energy metabolism via a pathway other than Complex V.

### DR Restores the Balance of the RhoA and ROCK Signaling Pathway During I/R

**FIGURE 10:**
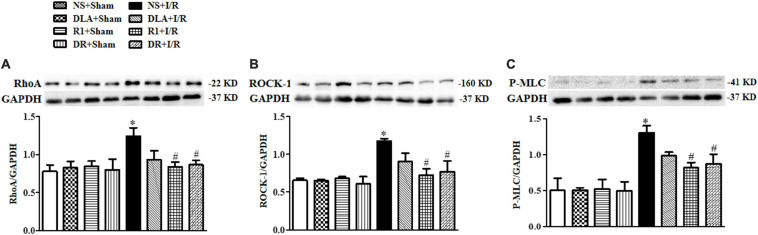
The effect of DLA, R1, and DR pretreatment on the level of Ras homolog gene family, member A (RhoA), Rho-associated kinase (ROCK), and phosphorylated myosin light chain (P-MLC) in rat heart tissue after I/R for 90 min. **(A)** Examples of Western blot bands of RhoA in various groups with quantification shown below. Quantitative results for RhoA band intensities were normalized to GAPDH (n = 6). **(B)** Examples of Western blot bands of ROCK-1 in various groups with quantification shown below. Quantitative results for ROCK-1 band intensities were normalized to GAPDH (n = 6). **(C)** Examples of Western blot bands of P-MLC in various groups with quantitative results shown below. Quantitative results for P-MLC band intensities were normalized to GAPDH. Data are presented as means ± SEM (n = 6). *p < 0.05 vs. NS + Sham group; #*p* < 0.05 vs. NS + I/R group.

Ras homolog gene family, member A and ROCK signaling is known to be involved in various heart conditions, including myocardial I/R injury. We next assessed the level of RhoA, ROCK-1, and P-MLC in different groups to learn if this signaling is implicated in the role of the drugs tested. As shown in Figure 10, the levels of RhoA, ROCK-1, and P-MLC were notably elevated after I/R, compared with the sham groups, which were restrained by R1 and DR pretreatment. On the other hand, DLA did not show any effect on any of the proteins examined.

## Discussion

The present study demonstrated that DR, a combination of DLA and R1 in a proportion of 4:1, was more efficient than either DLA or R1 alone in protecting MBF from decline and attenuation of infarction after I/R. The changes in MBF and myocardium infarct are sequelae of a spectrum of insults that take place in response to I/R, most, if not all, of which result from the dysregulated mitochondrial respiratory chain. The observed effect of DR reflects the role of DLA and R1, both of which are known to interfere in the respiratory chain. On the other hand, when combining two herb extracts, each having unique activity, different types of interactions may occur, such as complementary, counteractive each other, or mutually reinforcing. We tested the interaction of DLA and R1 in DR in terms of the effect on some known targets of the two herb extracts. The results observed are listed in Table 1.

**TABLE 1:** The effect of 3,4-dihydroxyl-phenyl lactic acid (DLA), notoginsenoside R1 (R1), and a combination of 3,4-dihydroxyl-phenyl lactic acid and notoginsenoside R1 (DR) on the parameters tested.

	**DLA**	**R1**	**DR**
ATP/ADP	-	+	+
ATP/AMP	+	+	+
ATP 5D	-	+	+
Complex V activity	-	+	+
RhoA	-	+	+
ROCK-1	-	+	+
P-MLC	-	+	+
Caspase 3	-	-	-
Cleaved Caspase 3	+	+	+
Caspase 9	-	-	-
Cleaved Caspase 9	+	+	+
Bcl-2/BAX	+	+	+
MPO	+	-	+
MDA	+	+	+
8-OHdG	+	-	+
Complex I activity	+	-	+
NDUFA10	+	-	+
Sirt-1	+	-	+
Sirt-1 affinity	+	-	

*+, positive; -, negative.*

Myocardial apoptosis is an inherent consequence of cardiac I/R injury. Both intrinsic and extrinsic apoptotic signaling pathways are implicated in the initiation of myocardial apoptosis in I/R injury (Lopez-Neblina et al., 2005). We examined DLA, R1, and DR as to their effect on I/R-induced myocardial apoptosis, revealing that DLA and R1 are both able to attenuate the apoptosis after I/R, consistent with a previous report, with DLA being a little more potent. In view of the known role of both DLA and R1 in protecting against apoptosis, this result shows that the two medicines interact with each other complementarily in DR to exert an effect on apoptosis.

Oxidative stress takes place in response to the I/R challenge, which results in the damage of biomacromolecules as well as apoptosis (Kurian et al., 2016). We examined the effect of DLA, R1, and DR on the level of MPO, MDA, and 8-OHdG, the oxidative stress and damage markers, in the myocardium tissue. The results showed that DLA and DR exhibited a similarly protecting role in reducing MPO and 8-OHdG after I/R, while R1 had no effect, suggesting DLA as a potent medicine for the prevention of leukocyte infiltration and inflammation caused by I/R, although DLA, R1, and DR exhibited a similarly protecting role in reducing MDA.

**FIGURE 11:**
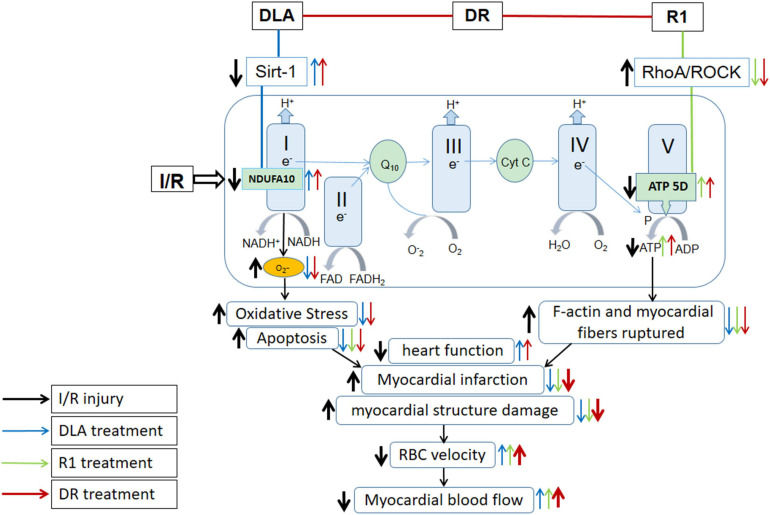
Summary of the effect of DR on myocardial I/R injury and the signaling pathways implicated. DR with DLA and R1 as two ingredients exerted effect by modulating mitochondrial respiratory chain Complex I and V, respectively, thus revealing a better outcome than DLA or R1 alone in TTC, BBC velocity, and MBF, which are the manifestations of a spectrum of insults.

Energy metabolism disturbance is a critical event in the pathogenesis of myocardial I/R injury, which contributes to a range of insults after I/R, such as rupture of F-actin cytoskeleton in cardiomyocytes, apoptosis, and oxidative stress, among others (Han et al., 2019). As previously reported, we observed a beneficial role of DLA, but not R1, in the protection of the downregulation of Sirt-1 and NDUFA10 and the activity of mitochondrial Complex I after I/R, while a protective effect of R1, but not DLA, on mitochondrial Complex V dysregulation. Noticeably, DR exhibits a role of both DLA and R1 with efficiency nearly identical to their each alone, resulting in a better outcome in 2,3,5-triphenyltetrazolium chloride (TTC), RBC, and MBF, all of which are the consequence of a spectrum of insults (Figure 11). The role of DLA in protecting the downregulation of Sirt-1 is most possibly accounted for by its binding to Sirt-1. This opinion is sustained by the fact that R1 failed to bind to Sirt-1 and was unable to prevent Sirt-1 downregulation as well. The mechanism underlying the effect of DLA or DR binding on Sirt-1 expression is, at present, unclear and required to be clarified by more studies.

Ras homolog gene family, member A/Rho-associated kinase signaling is a pathway known to participate in the pathogenesis of I/R injury (Yan et al., 2018). The present study revealed that DLA alone at the current dosage did not show any effect on the upregulated RhoA/ROCK signaling after I/R, while R1 showed an effect on the increased RhoA, ROCK-1, and P-MLC expression. DR played a role in downregulating RhoA/ROCK-1/P-MLC similarly to R1. This result suggests that R1 in DR takes responsibility for regulating the RhoA/ROCK signaling pathway, and the supplement of DLA did not interfere in the effect of R1 on this signaling.

Myocardial blood flow is determined by a number of variables, including the number of open vessels and RBC velocity. In the present study, MBF was assessed by a Laser-Doppler Perfusion Imager, which scanned an area covering the infarct zone, the penumbra, as well as the normal myocardium. In view of the interruption of blood flow in the infarct zone, the infarct size will be the dominant determinant of MBF. This is why the results of MBF in the present study are in line with that of TTC staining. In addition, assessment of heart function in the present study showed that DR, DLA, as well as R1 improved the heart function to some extent at 90 min after reperfusion with the effect of DR similar to that of DLA but more potent than that of R1. This result is not completely consistent with what was predicted from the result of TTC staining. The reason for this inconsistency is, at present, unclear and needs to be clarified by further study.

## Conclusion

(1) This study showed that DLA attenuated the downregulated expression of Sirt-1, NDUFA10, and inactivity of Complex I, and thus relieved oxidative stress and apoptosis, but had no potential to inhibit the activation of RohA/ROCK and elevate ATP 5D and ATP content, a factor responsible for the integrity of F-actin and myocardial fiber. R1 inhibited the RohA/ROCK activity, upregulated the ATP 5D expression, and increased the myocardial ATP content, alleviating myocardial F-actin and myocardial fiber rupture, while it had no effect on the downregulated expression of Sirt-1 and NDUFA10 and the inactivity of Complex I.
(2) As a combination of DLA and R1, DR revealed a protective effect on myocardial I/R injury by modulating both mitochondrial respiratory chain Complex I and V, presenting an integrated effect of DLA and R1 (Figure 11).
(3) For some of the endpoints tested, such as MBF, infarct area/AAR, and venous RBC velocity, the effect of DR was more potent than that of DLA or R1 alone.

### Perspective

Percutaneous coronary intervention is currently used for IHD, one of the major threats to human life worldwide. However, PCI frequently results in reperfusion injury. This study explored the role of DR, a composite of DLA and R1, in the protection of ischemia and reperfusion injury and the underlying mechanism. DR significantly attenuated I/R-induced infarct size and myocardial microcirculatory disturbance. Moreover, it ameliorated I/R-induced F-actin rupture, apoptosis, and oxidative stress. The effect of DR was attributable to its two components, with DLA binding to Sirt-1 to regulate the activity of Complex I as an antioxidant, while R1 regulating ATP 5D expression to enhance the activity of Complex V to attenuate energy metabolism disorder.

## Data Availability Statement

The raw data supporting the conclusion of this article will be made available by the authors, without undue reservation, to any qualified researcher.

## Ethics Statement

All experimental protocols were approved by Peking University Biomedical Ethics Committee Experimental Animal Ethics Branch (LA2010-001), complying with the Guide of Peking University Animal Research Committee.

## Author Contributions

LY performed the research, analyzed the data, and wrote the manuscript. C-SP contributed to the western blotting. Y-YL and JL contributed to the animal experiments. Y-CC contributed to the SPR experiments. B-HH and XC contributed to the electron microscopy. X-HW and PH contributed to morphology and immunofluorescence. J-YF revised the manuscript. QL, KS, L-LY, and KH contributed to the other experiments. J-YH designed and funded the research, interpreted the data, and finally approved the submission of this manuscript. All authors read and agreed with the manuscript.

## Conflict of Interest

The authors declare that the research was conducted in the absence of any commercial or financial relationships that could be construed as a potential conflict of interest.

## Abbreviations

DLA: 3,4-dihydroxyl-phenyl lactic acid
R1: notoginsenoside R1
DR: a combination of 3,4-dihydroxyl-phenyl lactic acid and notoginsenoside R1
MBF: myocardial blood flow
RBC: red blood cell velocity
SPR: surface plasmon resonance
I/R: ischemia reperfusion
NS: normal saline
ROS: reactive oxygen species
TUNEL: terminal deoxynucleotidyl transferase-mediated dUTP-biotin nick end-labeling
TTC: 2,3,5-triphenyltetrazolium chloride
HE: hematoxylin-eosin
IHD: ischemic heart disease
PCI: percutaneous coronary intervention
ETC: electron transport chain
NDUFA10: NADH dehydrogenase (ubiquinone) 1 alpha subcomplex 10
Sirt-1: Sirtuin1
RhoA, Ras homolog gene family: member A
ROCK: Rho-associated kinase
ATP 5D: ATP synthase δ subunit
Complex I: the mitochondrial Complex I
Complex V: the mitochondrial Complex V
F-actin: filamentous actin
ATP: adenosine triphosphate
ADP: adenosine diphosphate
AMP: adenosine monophosphate
P-MLC: phosphorylated myosin light chain
MPO: myeloperoxidase
MDA: malondialdehyde
8-OHdG: 8-oxo-deoxyguanosine
Bax: Bcl-2 associated X protein
Bcl-2: B-cell lymphoma 2
Caspase 3/9: cysteinyl aspartate specific proteinase 3/9
LV: left ventricle
HR: heart rate
LVSP: left ventricular systolic pressure
LVDP: left ventricular diastolic pressure
+dp/dtmax: left ventricular maximum upstroke velocity
–dp/dtmax: left ventricular maximum descent velocity
